# Prognostic value of neutrophil-to-lymphocyte ratio in patients with non–muscle-invasive bladder cancer with intravesical Bacillus Calmette–Guérin immunotherapy: a systematic review and meta-analysis

**DOI:** 10.3389/fimmu.2024.1464635

**Published:** 2024-10-23

**Authors:** Jiaguo Huang, Li Lin, Dikai Mao, Runmiao Hua, Feifei Guan

**Affiliations:** ^1^ Department of Urology, Affiliated Xiaoshan Hospital, Hangzhou Normal University, Hangzhou, China; ^2^ Department of Science and Education, Affiliated Xiaoshan Hospital, Hangzhou Normal University, Hangzhou, China; ^3^ Physical Examination Center, Affiliated Xiaoshan Hospital, Hangzhou Normal University, Hangzhou, China

**Keywords:** non-muscle-invasive bladder cancer, bacillus Calmette-Guérin, neutrophil-tolymphocyte ratio, recurrence, progression, systematic review, meta-analysis

## Abstract

**Background:**

The predictive accuracy of the preoperative neutrophil-to-lymphocyte ratio (NLR) on the prognosis of patients with non-muscle-invasive bladder cancer (NMIBC) with intravesical Bacillus Calmette–Guérin immunotherapy (BCG) after transurethral resection of the bladder tumor (TURBT) remains unknown. Therefore, the current study performed a systematic review and meta-analysis to examine the relationship between preoperative NLR and the prognosis of patients with NMIBC with intravesical BCG immunotherapy.

**Methods:**

For this systematic review and meta-analysis, articles were retrieved from PubMed, Cochrane Library, Web of Science, and Embase databases from their inception to 14 May 2024. The role of NLR in predicting recurrence and progression in NMIBC was determined using pooled hazard ratios (HRs) and 95% confidence intervals (CIs).

**Results:**

Seven articles were included in this meta-analysis, involving 4,187 patients. An elevated NLR was significantly associated with recurrence (HR = 2.67, 95% CI = 1.34–5.32, *P* < 0.001) and progression (HR = 1.72, 95% CI = 1.13–2.60, *P* = 0.004) in patients with NMIBC with intravesical BCG immunotherapy.

**Conclusion:**

This meta-analysis demonstrated that elevated preoperative NLR levels were significantly associated with recurrence and disease progression in patients with NMIBC who underwent intravesical BCG immunotherapy after TURBT.

**Systematic review registration:**

https://inplasy.com/inplasy-2024-7-0058/, identifier 202470058.

## Introduction

Bladder cancer (BCa) is one of the most commonly diagnosed malignancies worldwide and the most common malignancy of the urologic system, with approximately 573,000 new cases and 213,000 deaths according to the Global Cancer Statistics 2020 (1). On the basis of the depth of tumor invasion, BCa can be classified as non–muscle-invasive bladder cancer (NMIBC) and muscle-invasive bladder cancer (MIBC). In approximately 70% of the patients diagnosed with BCa, the disease is limited to the mucosa [stage Ta, carcinoma *in situ* (CIS)] or submucosa (stage T1) (2, 3). The gold standard for the treatment of NMIBC is transurethral resection of the bladder tumor (TURBT) followed by intravesical instillation therapy and then undergoing a second surgery if necessary (3). Bacillus Calmette–Guérin (BCG) has been shown to be the most effective intravesical immunotherapy for preventing the recurrence of NMIBC by inducing an immune response in the bladder to attack cancer cells (4–7). However, decades have passed since BCG was suggested as a treatment for NMIBC, but immunotherapy for NMIBC has not progressed much (8). The intravesical BCG immunotherapy is based on an attenuated non-pathogenic strain of Mycobacterium bovis that was originally used as a vaccine against tuberculosis (9–11). NMIBC typically shows a favorable prognosis with a 5-year overall survival (OS) rate of approximately 90%; still 30%–80% of cases exhibit recurrences and 45% of cases progress to muscle invasion were observed within 5 years (12). The clinical results of intravesical BCG immunotherapy are promising, but a subset of patients has shown unwanted results, such as the lack of clinical response, tumor recurrence, and tumor progression (13). Therefore, predicting BCG failure, tumor recurrence, and progression may facilitate timely radical cystectomy or combination therapy and improve the survival rate of patients. Currently, the European Organization for Research and Treatment of Cancer (EORTC) model is widely utilized to assess the risk of recurrence and progression of NMIBC treated with intravesical BCG immunotherapy. The scoring system has proven useful in clinical practice, but the accuracy of the EORTC model requires further improvement (14, 15). At present, other personalized models and optimization schemes have been proposed, but their performance has not been clinically verified (16, 17). One approach to address this challenge is to optimize the general treatment, particularly the initial patient condition (18). Consequently, easily accessible and objective predictors are needed to improve the risk classification and prediction of recurrence and progression of NMIBC.

Several studies have suggested that some inflammatory markers may be associated with the prognosis of intravesical BCG immunotherapy for NMIBC. The neutrophil-to-lymphocyte ratio (NLR) is a biomarker for assessing inflammatory status and is easily obtainable in clinical practice (19). Many systematic reviews and meta-analyses have effectively integrated the findings of multiple studies, yielding valuable insights into the complex relationships between NLR and solid tumors [e.g., breast cancer (20), cervical cancer (21), esophageal cancer (22), colorectal cancer (23), prostate cancer (24), and pancreatic cancer (25)]. The evidence consistently suggests that elevated NLR levels are significantly associated with poor prognosis in patients who develop these specific cancer types. Furthermore, NLR levels have been found to be independent predictors of the prognosis of BCa. In a meta-analysis of 17 studies reported by Xingxing Tang et al., elevated NLR was found to predict poor clinical outcomes in patients with BCa, including OS, recurrence-free survival (RFS), progression-free survival (PFS), and cancer-specific survival (CSS) (26). Another meta-analysis of six studies confirmed that an elevated preoperative NLR predicted worse RFS and PFS in patients with NMIBC treated with TURBT (27).

Despite previous studies having reported the relationship between NLR and survival outcomes in patients with NMIBC, no comprehensive meta-analysis has been conducted on patients with NMIBC with intravesical BCG immunotherapy. Patients receiving intravesical BCG immunotherapy after TURBT represent independent treatment types, and several studies have reported that preoperative NLR levels can predict the efficacy of intravesical BCG immunotherapy and disease relapse or progression in patients with NMIBC. Based on these previous findings, this meta-analysis aimed to systematically evaluate the relationship between preoperative NLR and the prognostic value of postoperative intravesical BCG immunotherapy in patients with NMIBC.

## Materials and methods

### Study registration

The present systematic review and meta-analysis was conducted on the basis of the Preferred Reporting Items for Systematic Reviews and Meta-Analyses guideline (28). The protocol of this review has been registered online on INPLASY (202470058).

### Search strategy

Relevant studies were systematically searched on PubMed, Cochrane Library, Web of Science, and Embase databases from inception to 14 May 2024, with no language limitations. The search terms combined the suggested words by Medical Subject Heading (MeSH) with other related words. The search query in PubMed was as follows: ((((Neutrophil-to-Lymphocyte Ratio[Title/Abstract]) OR (Neutrophil to Lymphocyte Ratio[Title/Abstract])) OR (NLR[Title/Abstract])) AND (((((((((Non-Muscle Invasive Bladder Neoplasms[MeSH Terms]) OR (Non Muscle Invasive Bladder Neoplasms[Title/Abstract])) OR (NMIBC[Title/Abstract])) OR (Non-Muscle-Invasive Bladder Cancer[Title/Abstract])) OR (Bladder Cancer, Non-Muscle-Invasive[Title/Abstract])) OR (Bladder Cancers, Non-Muscle-Invasive[Title/Abstract])) OR (Cancer, Non-Muscle-Invasive Bladder[Title/Abstract])) OR (Cancers, Non-Muscle-Invasive Bladder[Title/Abstract])) OR (Non-Muscle Invasive Bladder Cancer[Title/Abstract]))) AND (((((Bacillus Calmette-Guerin[Title/Abstract]) OR (Bacillus Calmette Guerin[Title/Abstract])) OR (Bacillus Calmette-Guérin[Title/Abstract])) OR (Bacillus Calmette Guérin[Title/Abstract])) OR (BCG[Title/Abstract])). Additional relevant research was identified by manually reviewing the references cited in the captured articles.

### Inclusion and exclusion criteria

Studies were included if they met the following criteria: (1) articles reporting the relationship between NLR and the prognosis of patients with NMIBC receiving intravesical BCG immunotherapy after surgery, using the formula NLR = neutrophil count/lymphocyte count; (2) groups were classified according to their preoperative NLR levels; (3) specific endpoints of interest, including recurrence and progression; and (4) availability of hazard ratios (HRs) or odds ratios (ORs) with 95% confidence intervals (CIs) or the ability to calculate them from the data presented in the articles. The exclusion criteria consisted of the following: (1) case reports, conference abstracts, editorials, and reviews; (2) studies with insufficient information and extractable data on the treatment and main outcomes of patients with NMIBC; (3) animal studies; (4) duplicates, or surveys investigating the same sample; and (5) the full text was unavailable.

### Study selection and data extraction

In order to determine suitability of the articles, the titles and abstracts screened were independently by two investigators (JH and LL). Subsequently, the full texts were examined. Non-English articles were translated using Google Translate if necessary. In cases of replicated publications, the study with the most extensive information was considered. Discrepancies in study inclusion between the two independent investigators were settled by consulting a third party (DM). Data from the articles were extracted and recorded in a Microsoft Excel spreadsheet (Microsoft Corporation, Redmond, WA) by two investigators (JH and LL) independently, including the following: the name of first author; the year of study publication; study region (country); number of study centers; mean or median age; sample size; tumor stage; follow-up time (months); NLR cutoff; cutoff selection; and survival outcome, HRs, or ORs with 95% CIs.

### Quality assessment

Two investigators (JH and LL) were involved in the quality evaluation using the Newcastle–Ottawa Scale (NOS), and a third party (DM) acted as adjudicator in case of disagreement. A higher score indicated a higher study quality, showing a positive correlation between the two. Studies with scores higher than 6 were considered of high quality.

### Study outcomes and statistical analysis

Meta-analysis was conducted using Stata 16.0 software (StataCorp). HRs and 95% CIs were computed as the combined effect size to assess the relationship between pre-treatment NLR and recurrence and progression. The Q test was used to analyze the heterogeneity among the results of the included studies. *I* ^2^ was utilized to quantitatively assess the magnitude of heterogeneity. Based on *I* ^2^ statistics, heterogeneity was categorized as high (above 75%), moderate (25% to 75%), and low (below 25%) (29). When *I* ^2^ >50% and/or *P* < 0.05, the random model was used; otherwise, the fixed model was used. Potential sources of heterogeneity were identified through subgroup analyses. If grouped data on subgroup categories were not available, then they were excluded. In addition, the meta-analysis was also subjected to a sensitivity analysis in order to eliminate the effect of individual study data on survival outcomes. The publication bias was evaluated using Egger’s test. *P* < 0.05 was regarded statistically significant.

## Results

**Figure 1:**
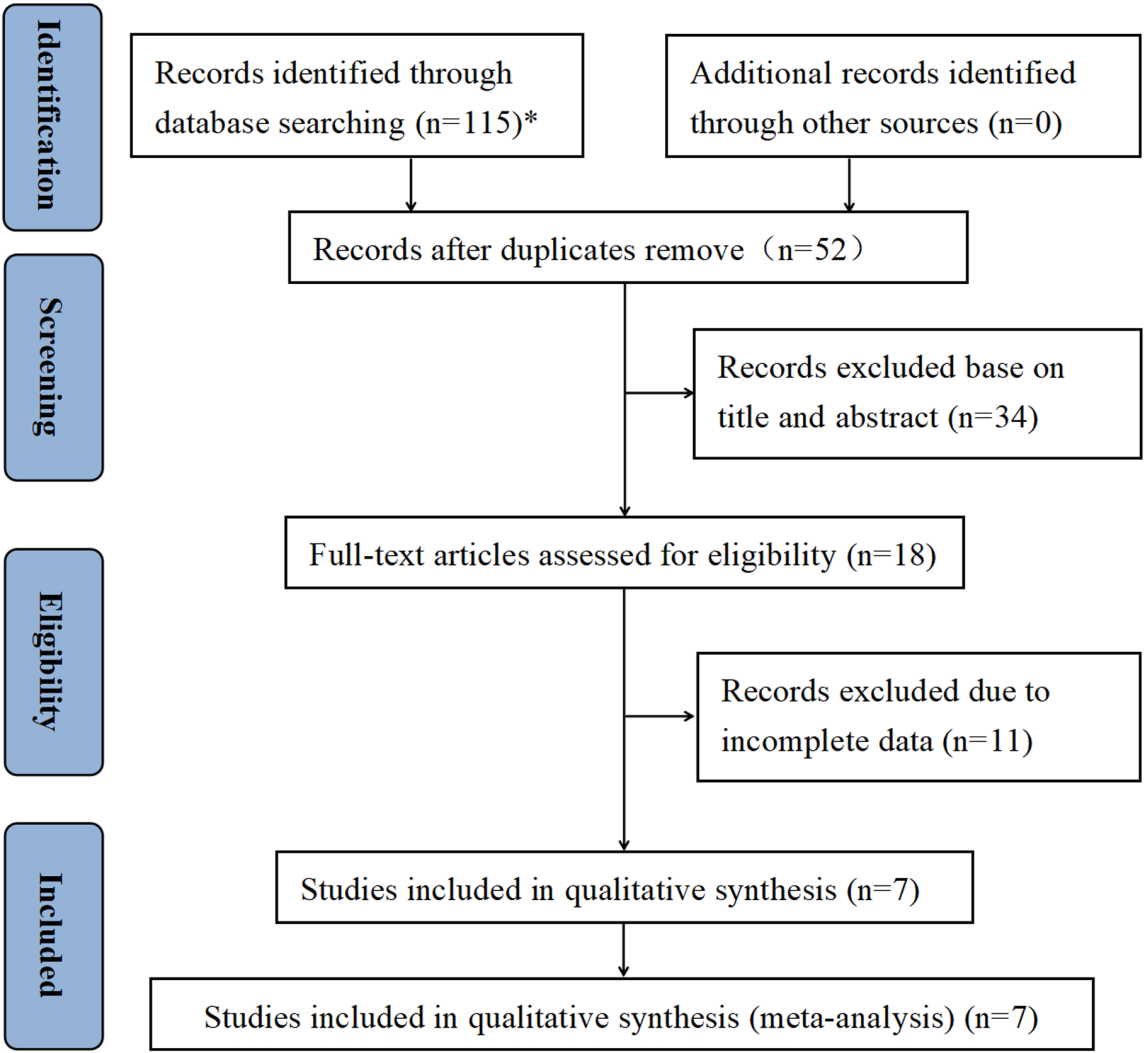
PRISMA diagram showing the study selection process. *PubMed (n = 31), Web of Science (n = 39), Cochrane Library (n = 1), and Embase (n = 44).

As a whole, 115 publications were retrieved by searching PubMed (n = 31), Web of Science (n = 39), Cochrane Library (n = 1), and Embase (n = 44). After removing duplicate articles, 52 articles remained for further screening. After reading the titles and abstracts of these publications, 18 studies were identified for full-text screening. In the meta-analysis, a total of seven retrospective studies published between 2017 and 2023 and involving 4,187 participants were ultimately included (30–36). The Preferred Reporting Items for Systematic reviews and Meta-Analyses (PRISMA) flowchart of the study selection is described in **Figure 1**. Four studies were conducted in Asian, and five studies were conducted in non-Asian countries. The sample size of the included articles ranged from 89 to 1,709. In terms of tumor type, the seven studies focused on NMIBC and all were patients who underwent intravesical BCG immunotherapy after TURBT. The pathological stage was Ta and/or T1, and five studies reported concurrent CIS. The cutoff values for NLR ranged from 1.361 to 3. Furthermore, all seven studies evaluated the association of preoperative NLR with tumor recurrence, and six studies evaluated the association of NLR with tumor progression. All studies scored at least six points on the NOS, indicating a high overall quality. **Table 1** shows an overview of the baseline data and quality evaluations of the studies.

**Table 1:** An overview of the baseline data and quality evaluations of the included studies.

Study	Year	Region	Study design	Number of study centers	Sample size	Age* (years)	Tumor stage	Follow- up (months)	NLR cutoff	Cutoff selection	Survival outcome	HR	LCI	UCI	NOS score
Cai et al. (30)	2023	China	Retrospective	Unicenter	421	61.79 ± 11.51	Ta, CIS, and T1	17 (IQR, 12–27)	1.361	ROC	Recurrence	2.732	1.178	6.329	6
											Progression	1.767	0.619	5.051	
Camtosun et al. (31)	2017	Turkey	Retrospective	Unicenter	89	65 (range, 39–83)	Ta, CIS, and T1	28.7 (range, 12–78)	2.5	ROC	Recurrence	4.9	1.68	14.28	7
Chung et al. (32)	2021	Korea	Retrospective	Unicenter	281	67.38 ± 10.58	Ta and T1	46	2.29	ROC	Recurrence	2.514	1.657	3.483	7
											Progression	6.119	1.975	21.622	
D’Andrea et al. (33)	2017	Austria	Retrospective	Multicenter	110	NA	Ta and T1	68	3	ROC	Recurrence	1.6	0.6	4.7	7
											Progression	3.1	0.9	10.7	
Ferro et al. (34)	2022	Italy, Romania, and USA	Retrospective	Multicenter	1,382	69.87 ± 9.71	CIS and T1	27 (IQR, 8–35)	NA	NA	Recurrence	7.471	5.394	10.346	7
								22 (IQR, 36–58)			Progression	1.014	0.8	1.285	
Li et al. (35)	2023	China	Retrospective	Unicenter	195	64.17 ± 11.05	CIS and T1	30.18 ± 15.67	NA	NA	Recurrence	2.303	1.457	3.638	6
											Progression	2.334	1.34	4.066	
Nishikawa et al. 36	2023	Japan	Retrospective	Multicenter	1,709	72 (IQR, 65–78)	Ta, CIS, and T1	60	2.5	Based on reports	Recurrence	1.093	0.902	1.325	8
											Progression	1.283	0.95	1.732	

CIS, carcinoma *in situ*; HR, hazard ratio; IQR, interquartile range; LCI, lower confidence interval; NLR, neutrophil-to-lymphocyte ratio; NOS, Newcastle–Ottawa Scale; ROC, receiver operating characteristic curve; UCI, upper confidence interval.

NA, Not Available.*Age including mean age and/or median age.

### Association of preoperative NLR and recurrence in patients with NMIBC with intravesical BCG immunotherapy

**Figure 2:**
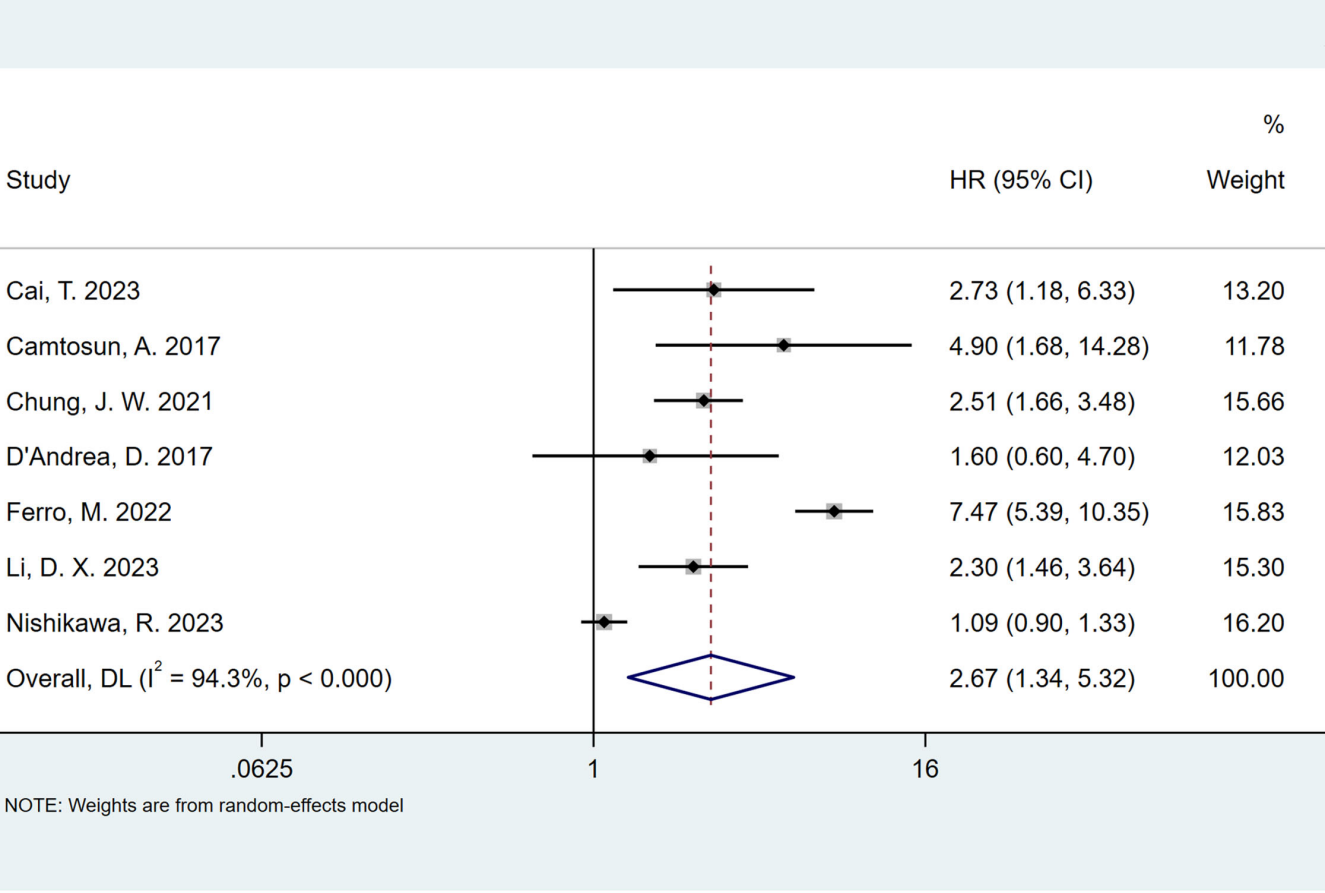
Forest plots of the prognostic role of NLR for recurrence in patients with NMIBC.

**Figure 3:**
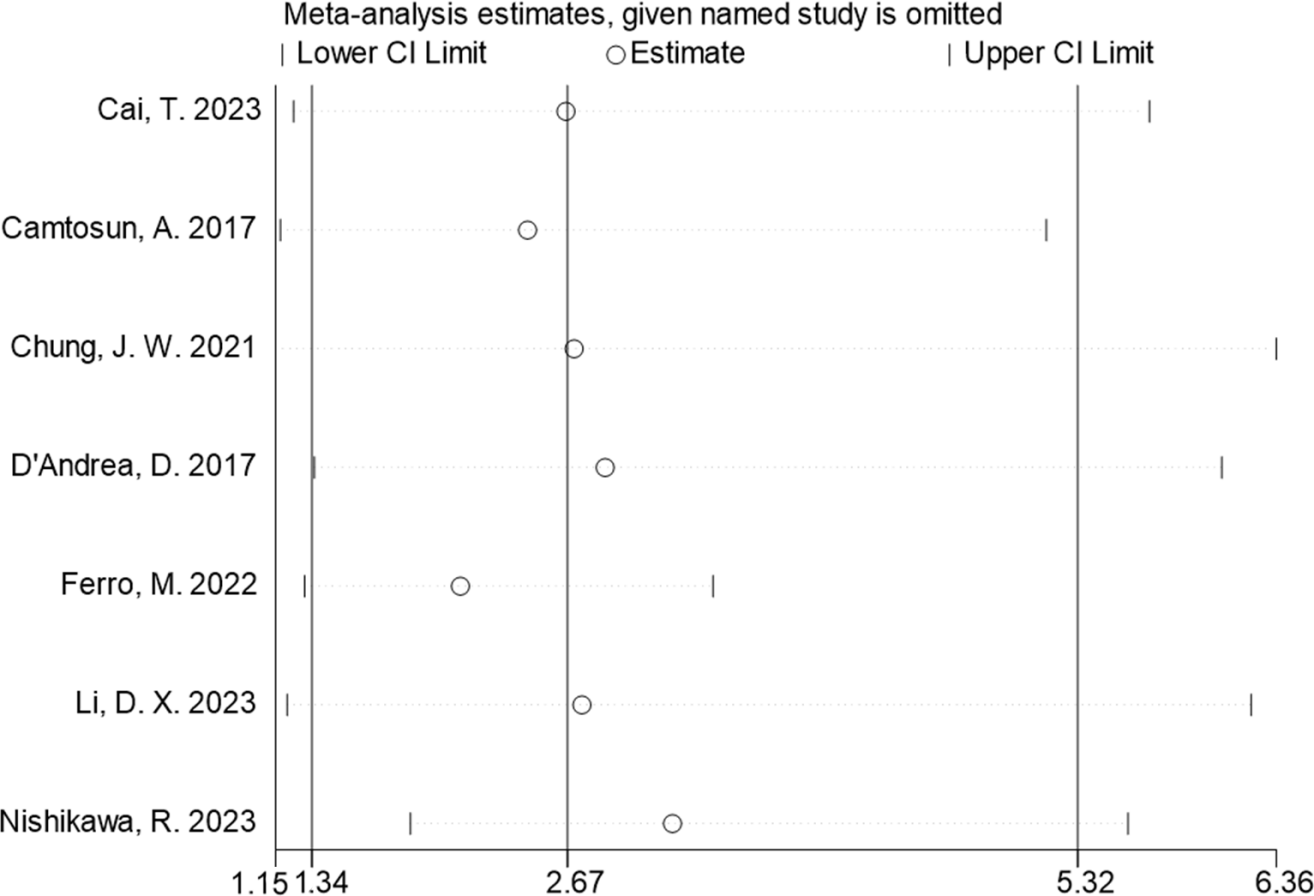
Sensitivity analyses for the association of NLR and recurrence in patients with NMIBC.

The seven studies all reported the predictive value of preoperative NLR in the risk of tumor recurrence. A random effects model (*I* ^2^ = 94.3%, *P* < 0.001) was employed and the pooled analysis showed that patients with elevated NLR tended to have a higher recurrence risk (HR = 2.67, 95% CI = 1.34–5.32) (**Figure 2**). The sensitivity analysis indicated that removing individual studies did not affect the results of this study; hence, the results of the above random effects were stable and reliable, as shown in **Figure 3**. Then, the source of heterogeneity was assessed on the basis of ethnicity (Asian and non-Asian), number of study centers (unicenter and multicenter), sample size (<300 subjects and ≥300 subjects), and tumor stage (with concomitant CIS and without concomitant CIS). Moreover, subgroup analyses demonstrated that preoperative NLR remained a significant prognostic factor for recurrence and failed to find the source of heterogeneity, as shown in **Table 2**. The subgroup analyses did not explain the source of heterogeneity. Subsequently, Egger’s tests were conducted to evaluate publication bias, showing no statistically significant publication bias (*P* = 0.355).

**Table 2:** Subgroup analyses for the prognostic role of NLR for recurrence and progression in patients with NMIBC.

Outcome	Subgroups		Number of studies	HR (95% CI)	*I* ^2^	*P* for heterogeneity
Recurrence	Ethnicity	Asian	4	1.95 (1.13–3.36)	86.8%	<0.001
		Non-Asian	3	4.24 (1.68–10.72)	75.2%	0.018
	Number of study centers	Unicenter	4	2.56 (1.97–3.34)	0	0.648
		Multicenter	3	2.39 (0.54–10.60)	98.0%	<0.001
	Sample size	<300	4	2.47 (1.89–3.23)	0	0.502
		≥300	3	2.81 (0.66–12.00)	98.0%	<0.001
	Tumor stage	Without concomitant CIS	2	2.39 (1.68–3.38)	0	0.418
		With concomitant CIS	5	2.96 (1.14–7.69)	96.2%	<0.001
Progression	Ethnicity	Asian	4	2.04 (1.16–3.61)	65.5%	0.033
		Non-Asian	2	1.49 (0.53–4.23)	66.9%	0.082
	Number of study centers	Unicenter	3	2.63 (1.49–4.46)	23.7%	0.269
		Multicenter	3	1.20 (0.88–1.64)	50.5%	0.133
	Sample size	<300	3	2.85 (1.75–4.63)	3.8%	0.354
		≥300	3	1.13 (0.93–1.38)	8.5%	0.335
	Tumor stage	Without concomitant CIS	2	4.41 (1.86–10.41)	0	0.439
		With concomitant CIS	4	1.37 (0.97–1.93)	63.3%	0.043

CIS, carcinoma *in situ*; HR, hazard ratio; NLR, neutrophil-to-lymphocyte ratio.

### Association of preoperative NLR and progression in patients with NMIBC with intravesical BCG immunotherapy

**Figure 4:**
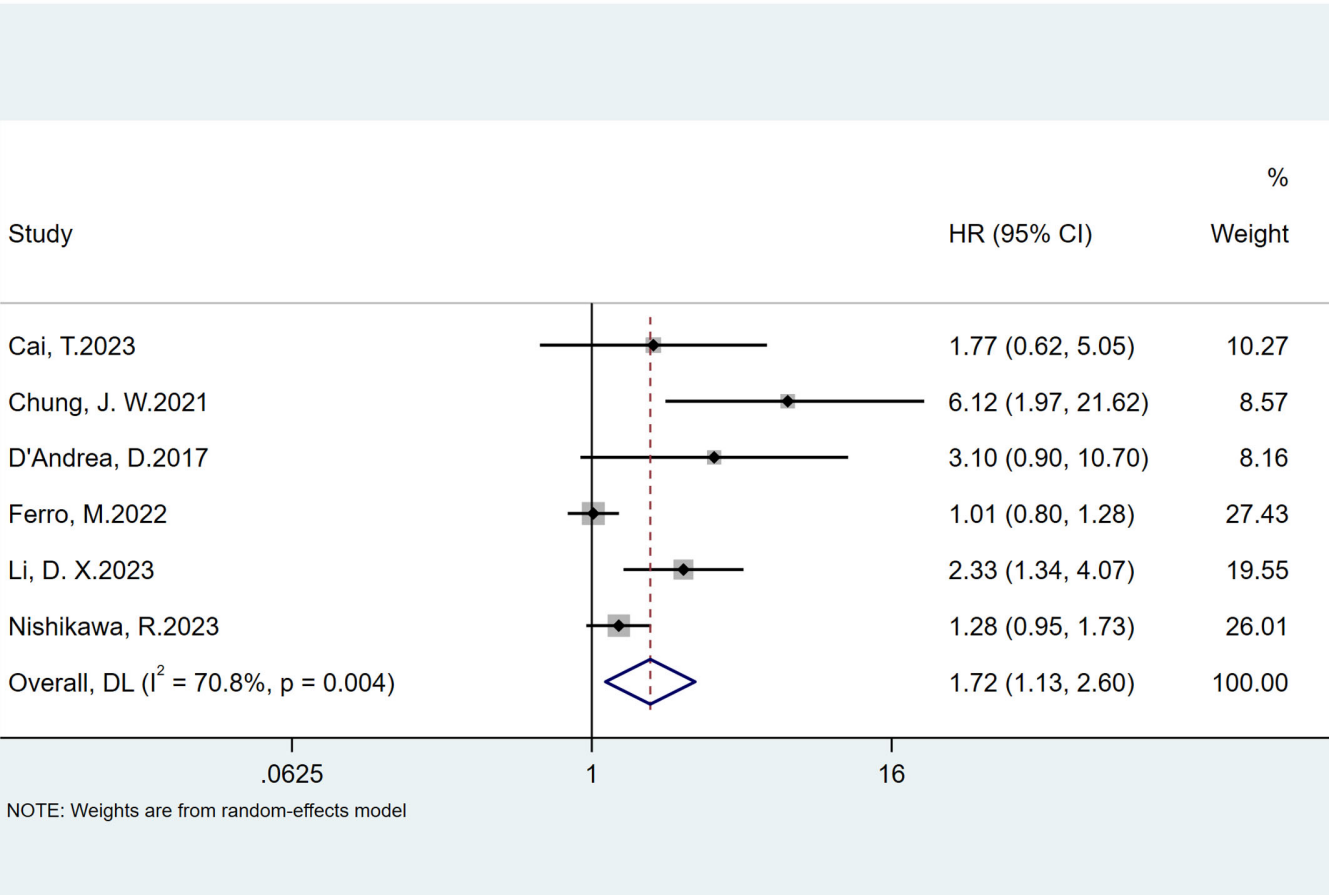
Forest plots of the prognostic role of NLR for progression in patients with NMIBC.

**Figure 5:**
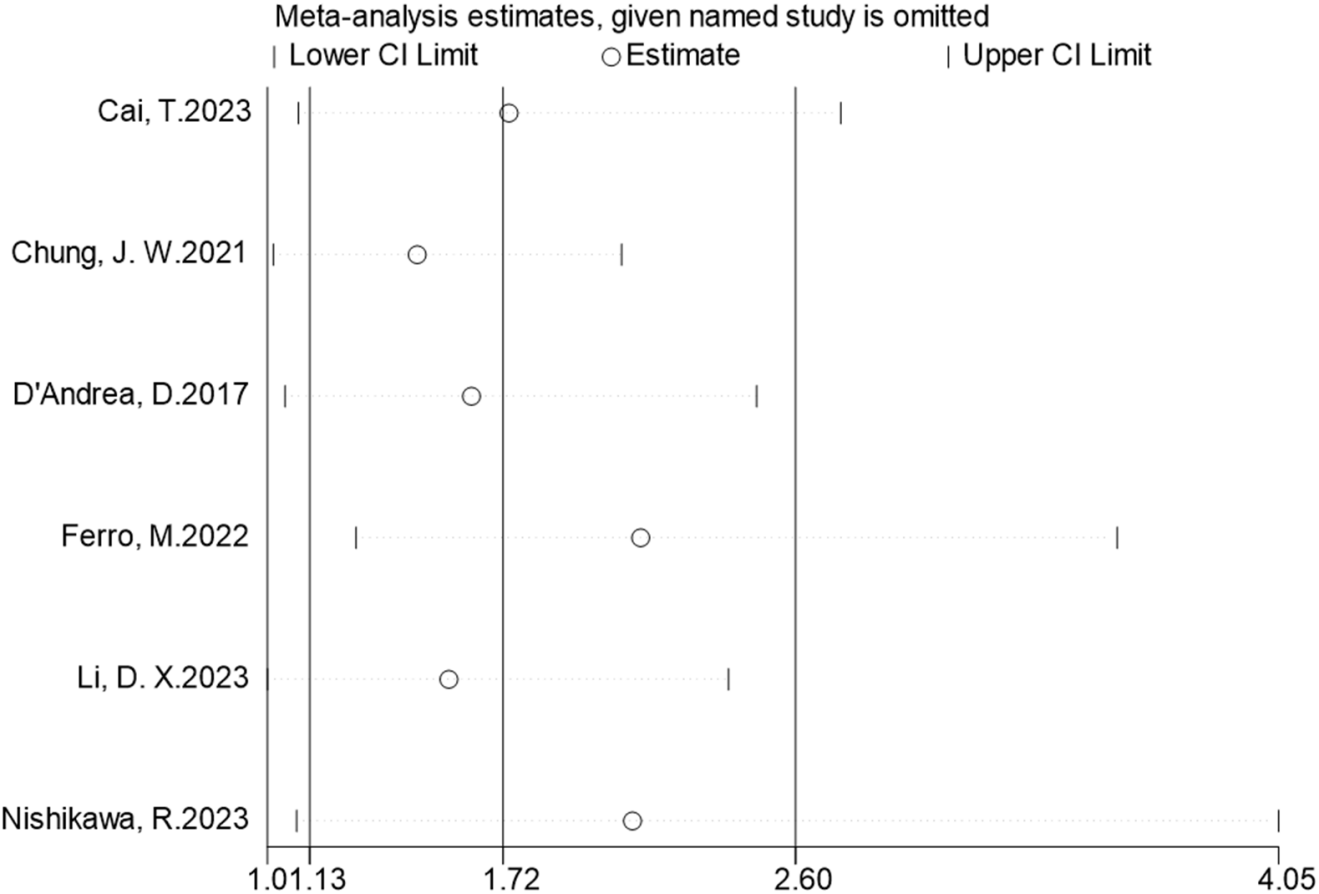
Sensitivity analyses for the association of NLR and progression in patients with NMIBC.

Six studies reported the predictive value of preoperative NLR in the risk of tumor progression; the pooled analysis showed that patients with elevated NLR tended to have a higher progression risk (HR = 1.72, 95% CI = 1.13–2.60), based on a random effects model (*I* ^2^ = 70.8%, *P* = 0.004) (**Figure 4**). The sensitivity analysis revealed that the arbitrary deletion of studies did not affect the results, indicating that the results of the above random effects are stable and reliable, as shown in **Figure 5**. Further subgroup analysis revealed that the number of study centers and the sample size might be the main source of heterogeneity (**Table 2**). Egger’s tests were conducted to evaluate publication bias, showing the possibility of publication bias (*P* = 0.023). However, discussing publication bias in a meta-analysis containing less than 10 studies presents a challenge (37). The number of studies with publication bias included in the above analysis was relatively small, so the trim-and-fill method cannot be used to assess publication bias.

## Discussion

Our research findings indicate that elevated preoperative NLR levels are a promising and cost-effective prognostic biomarker for recurrence and progression in patients with NMIBC with intravesical BCG immunotherapy. To the extent of our knowledge, this is the first meta-analysis to comprehensively examine the relationship between preoperative NLR levels and the risk of recurrence and progression in patients with NMIBC who underwent intravesical BCG immunotherapy. In patients with NMIBC receiving intravesical BCG immunotherapy after TURBT, the combined results showed a significant correlation between preoperative NLR and recurrence and progression. The increase in preoperative NLR levels independently increased the risk of recurrence and progression in patients with NMIBC receiving intravesical BCG immunotherapy after TURBT. These findings provide valuable insights into the potential of NLR as a prognostic marker for patients with NMIBC receiving intravesical BCG immunotherapy, which can guide the follow-up of patients with NMIBC.

TURBT and intravesical BCG immunotherapy are recommended as treatments for patients with intermediate- and high-risk NMIBC to reduce the risk of recurrence and progression of NMIBC (3, 38). The EORTC risk score is used to evaluate the risk of recurrence and progression of NMIBC receiving intravesical BCG immunotherapy after TURBT (15). Jae Wook Chung et al. reported that the combined use of preoperative NLR and EORTC systems could improve the predictive accuracy of disease progression (32). Hence, NLR represents a practical, cost-effective, and non-invasive biomarker that has been widely used to predict poor prognosis of tumors. The NLR was calculated using the formula below: NLR = neutrophil count/lymphocyte count. As a result, an elevated NLR could be caused by increased neutrophils and/or decreased lymphocytes. The predictive role of NLR in cancer can be explained by several potential mechanisms.

The development, invasion, proliferation, and metastasis of BCa could be promoted by many cell types. The microenvironment of cancer cells is altered by the recruitment and activation of stromal, adipocyte, fibroblast, inflammatory, and mesenchymal progenitors (39, 40). The potential predictive role of NLR in carcinogenesis and tumor invasiveness may be attributed to the interaction with other cell populations to produce cytokines and effector molecules as the number of neutrophils increases (41). In the tumor environment, neutrophils play a role in controlling immune responses by polarizing to antitumorigenic or protumorigenic phenotypes (42, 43). They are rapidly recruited to pathogen signals, including chemotactic mediators, lipid mediators, chemokines, and cytokines, to mediate host defense (44). The major pro-tumor activities include cancer cell proliferation, cell invasion, metastasis, extracellular matrix remodeling, angiogenesis, lymphangiogenesis, and suppression of antitumor immune surveillance (45). All of these contribute to tumor formation and development (46, 47). In addition, lymphocytes play a role in immune defense by promoting the death of cytotoxic cells and inhibiting the proliferation and migration of tumor cells (48, 49). Improvements in therapeutic outcomes are associated with lymphocyte infiltration in tumor tissues. In contrast, when lymphocyte numbers in the tumor microenvironment decrease, the anti-tumor ability decreases, leading to immune tolerance and tumor escape (50). Thus, elevated NLR levels resulting from elevated neutrophil count and decreased lymphocyte count are often associated with poor prognosis in patients with cancer. Consequently, patients with elevated NLR levels should receive more aggressive therapy and a closer follow-up to ensure the timely detection of tumor recurrence and progression. It is worth popularizing in clinical practice as a cost-effective and practical biomarker that could lead to a turning point in the treatment of patients with BCG-unresponsive NMIBC. The recommended treatment for BCG-unresponsive disease remains radical cystectomy and urinary diversion (3, 51). Therefore, to order to meet patients who desire a bladder-sparing approach or are too frail for major surgery, intravesical chemotherapy, chemo-hyperthermia, immunotherapy, inflammation-targeted agents, and gene therapy receive more attention; novel molecular therapeutic targets including Programmed Death-1 (PD-1)/Programmed Death-ligand 1 (PD-L1) and Cytotoxic T-lymphocyte-associated Protein 4 (CTLA-4) also receive attention (52–55).

Nonetheless, the relationship between the systemic NLR and the local bladder tumor infiltration of neutrophils and lymphocytes remains unknown. Wael Abdo Hassan et al. performed a pathological examination for tumor grade and stage and for tumor-infiltrating neutrophils and lymphocytes, and determined the NLR at the tissue level. They reported a significant increase in neutrophil count in high-grade BCa cases compared to that in low-grade cases. Moreover, a significant increase in neutrophil count and a decrease in CD8 lymphocytes were observed in MIBC cases compared to that in NMIBC cases, indicating the tumor-promoting effect of neutrophils and the possible role of CD8 lymphocytes in hindering the progression to muscle invasion. In addition, significantly higher NLR was found in MIBC cases compared to that in the NMIBC cases in high-grade neoplasms. NLR was more likely to be associated with the progression of tumor invasion compared to that in the tumor grade (56). The above findings indicated that the systemic NLR was correlated with tumor-infiltrating NLR at the tissue level. However, further study is required to confirm it.

However, there are several limitations that should be acknowledged in this meta-analysis. Firstly, because all the included studies were retrospective, confounding factors may affect the accuracy of our findings. Although the participants included were patients with NMIBC, there were differences in the basic characteristics, clinical stage, histological subtypes, and degree of tumor invasion and treatment of patients with NMIBC in the included literature. For example, there are obvious differences in the prognosis of different histological subtypes. According to previous literature reports, the risk of recurrence and progression of high-grade lesions is significantly higher than that of low-grade (57, 58). CIS is considered a precursor of invasive high-grade cancer, and 54% of patients will progress to myometrial invasion without treatment (59). The re-TURBT and upfront BCG may also affect the tumor prognosis (60, 61). According to the European Association of Urology (EAU) guidelines, lymph vessel invasion (LVI), CIS in prostatic urethra, and EAU risk groups also determined the different prognosis, but only one literature in the included studies mentioned LVI and CIS in prostatic urethra (58, 62, 63). Due to the relatively small sample size, all confounding factors could not be considered in the subgroup analysis. Further prospective studies should be conducted to validate the current findings and produce stronger evidence. Secondly, the criteria for choosing cutoff values differ across studies, which may introduce selection bias related to factors such as ethnicity or clinical setting. Careful consideration of standardized methods or cutoff values tailored to different patient populations or clinical settings has significant value. Thirdly, the publication bias needs to be considered. Finally, a significant heterogeneity in recurrence and progression was found. However, a subgroup analysis was performed and found a potential source of heterogeneity.

## Conclusion

This meta-analysis demonstrated that elevated preoperative NLR levels were significantly associated with recurrence and disease progression in patients with NMIBC who underwent intravesical BCG immunotherapy after TURBT. As a cost-effective and practical biomarker, NLR can serve as a useful tool to guide the management and follow-up of patients with NMIBC. However, further large-scale prospective validation studies are needed to confirm these findings.

## Data Availability

The original contributions presented in the study are included in the article/supplementary material. Further inquiries can be directed to the corresponding author.
